# The New York State Veterinary Medical Society

**Published:** 1893-02

**Authors:** 


### NEW YORK STATE VETERINARY MEDICAL SOCIETY

The following appointments are made:

*Committee on Constitution and By-Laws.*—Drs. R. A. McLean, Chairman, Brooklyn; W. L. Baker, Cortland; Jno. A. Bell, Watertown; C. D. Morris, Brooklyn; L. E. Willyoung, Albion.

*Committee on Legislation.*—Drs. C. D. Morris, Chairman, Brooklyn; Jas. Carnrite, Amsterdam; Prof. Jas. Law, Ithaca; Drs. R. A. McLean, Brooklyn; A. Drinkwater, Rochester; H. Sutterby, Batavia; N. P. Hinkley, Buffalo; W. L. Baker, Cortland.

*Committee on Publication.*—Drs. N. P. Hinkley, Chairman, Buffalo; J. M. Chase, Seneca Falls; Wilson Huff, Rome.

*Committee on Arrangements.*—Drs. N. P. Hinkley, Chairman, Buffalo; E. J. McLeod, Buffalo; Saml. Somerville, Jr., Buffalo;

L. A. Robinson, Buffalo; Jno. Wende, Buffalo; E. A. Wieland, Buffalo; Geo. E. Gangloff, Buffalo; Wm. Kirk, Niagara Falls, N. Y.

*Committee on Prosecution.*—Drs. John Wende, Chairman, Buffalo; H. Sutterby, Batavia; N. P. Hinkley, Buffalo.

COUNTY SECRETARIES.

| COUNTY. | SECRETARY. | RESIDENCE. |
|---|---|---|
| Schenectady, | R. D. Austin, | Schenectady. |
| Seneca, | J. M. Chase, | Seneca Falls. |
| Cayuga, | Geo. Gowland, | Auburn. |
| New York, | Thomas Giffen, | New York. |
| Steuben, | Frank Sutterby, | Bath. |
| Niagara, | Wm. Kirk, | Niagara Falls. |
| Orleans, | L. E. Willyoung, | Albion. |
| Genesee, | H Sutterby, | Batavia. |
| Erie, | E. J. McLeod, | Buffalo. |
| Chemung, | G. P. Jeffrey, | Elmira. |
| Cortland, | W. L. Baker, | Cortland. |
| Onondaga, | H. B. Crandall, | Syracuse. |
| Wayne, | W. S. Corlis, | Sodus. |
| Monroe, | A. Drinkwater, | Rochester. |
| St. Lawrence, | Chas. Cowie, | Ogdensburg. |
| Jefferson, | Jno. A. Bell, | Watertown. |
| Kings, | C. D. Morris, | Brooklyn. |
| Rensselaer, | H. McWhinnie, | Troy. |
| Oneida, | Wilson Huff, | Rome. |
| Oswego, | M. M. Poucher, | Oswego. |
| Montgomery, | Jas. Carnrite, | Amsterdam. |
| Herkimer, | D. H. Rowe, | Little Falls. |
| Otesego, | V. L. James, | Cooperstown. |
| Schuyler, | A. L. Hunter, | Watkins. |
| Tompkins, | J. A. Geming, | Ithaca. |
| Ontario, | W. G. Dodds, | Canandaigua. |

